# Chorioallantoic membrane (CAM) assay to study treatment effects in diffuse intrinsic pontine glioma

**DOI:** 10.1371/journal.pone.0263822

**Published:** 2022-02-14

**Authors:** Erica A. Power, Jenelys Fernandez-Torres, Liang Zhang, Ruiyi Yaun, Fabrice Lucien, David J. Daniels

**Affiliations:** 1 Department of Neurologic Surgery, Mayo Clinic, Rochester, MN, United States of America; 2 Mayo Clinic Graduate School of Biomedical Sciences, Rochester, MN, United States of America; 3 Department of Urology, Mayo Clinic, Rochester, MN, United States of America; 4 Department of Molecular Pharmacology and Experimental Therapeutics, Mayo Clinic, Rochester, MN, United States of America; Keio University School of Medicine Graduate School of Medicine: Keio Gijuku Daigaku Igakubu Daigakuin Igaku Kenkyuka, JAPAN

## Abstract

Diffuse intrinsic pontine glioma (DIPG) is a lethal pediatric brain tumor. While there are a number of *in vivo* rodent models for evaluating tumor biology and response to therapy, these models require significant time and resources. Here, we established the chick-embryo chorioallantoic (CAM) assay as an affordable and time efficient xenograft model for testing a variety of treatment approaches for DIPG. We found that patient-derived DIPG tumors develop in the CAM and maintain the same genetic and epigenetic characteristics of native DIPG tumors. We monitored tumor response to pharmaco- and radiation therapy by 3-D ultrasound volumetric and vasculature analysis. In this study, we established and validated the CAM model as a potential intermediate xenograft model for DIPG and its use for testing novel treatment approaches that include pharmacotherapy or radiation.

## Introduction

Diffuse midline gliomas harboring the H3K27M mutation, historically referred to as diffuse intrinsic pontine gliomas (DIPG) are rare and aggressive brain tumors predominately found in children [1, 2]. With a median survival of 12 months upon diagnosis, this tumor is one of the leading causes of cancer-related morbidity and mortality in children [3]. While countless clinical trials have failed to show any therapeutic benefit, recent advances in DIPG biology have shown that a majority of DIPG tumors have a mutation in a histone H3 gene resulting in a methionine (M) in place of lysine (K) at position 27 (H3K27M) [4–6]. The H3K27M mutation results in specific epigenetic changes throughout the H3 proteins including trimethylation of lysine (K) sites, and phosphorylation of serine (S) sites [7–9]. The most prominent epigenetic change in H3K27M tumors is the global reduction of H3K27 trimethylation and is thought to be one of the main drivers of tumorigenesis [7, 10–12]. With this knowledge, the World Health Organization (WHO) reclassified DIPGs to include H3K27M mutation status as well as the signature loss of trimethylation

Preclinical evaluation of novel therapeutics and treatments for this disease have relied on *in vitro* assays and *in vivo* rodent studies. Translation into the clinic relies on strong *in vivo* data that corresponds with the *in vitro* results, and this has been one of the hurdles in developing effective therapy for this disease. Recently, the development of good H3K27M *in vivo* rodent models has increased due to an increase in tumor samples and advancement in GEMM technologies [2, 13–15]. However, *in vivo* model development and consequential studies have been limited by the high demand for costly resources as well as time—most of the patient derived xenografts grow very slow in rodents [14].

Previous studies in other cancers have shown the chicken embryo Chorioallantoic Membrane (CAM) model is an affordable and time-efficient xenograft model for studying tumor biology and response to treatments [16–18]. Due to a lack of an immune system, patient derived cancer cells can be engrafted on to the chorioallantoic membrane of a chicken embryo. The CAM is a rich vascular network connected to embryonic circulation formed by the fusion of the allantois and chorion mesoderm layers where engrafted tumors can be subjected to various anti-cancer treatments [18]. Unlike other *in vivo* models, which can take months for a tumor to engraft and subsequent treatment to ensue, the CAM model only takes a couple of weeks and thus has a very desirable time frame. The aim of this study was to develop the CAM model for DIPG and assess its applicability for a variety of treatment strategies including drug and radiation treatment.

## Methods

### Cell culture

DIPGIV (H3.1K27M mutated), DIPGXIIIp* (H3.3K27M mutated) are patient-derived cell lines gifted to us by Dr. Michelle Monje (Stanford University) infected with CMV-GFP-Luc (also gifted by Dr. Monje). The patients from which these cell lines are derived are passed and their characteristics published by Dr. Monje [19]. Cells were cultured in media hormone mix serum-free complete media (MHM+++) which consisted of DMEM/F12 (Gibco), 25 mM glucose (Sigma), sodium bicarbonate (Gibco), 2mM glutamine (Gibco), HEPES (Gibco), penicillin/streptomycin (Gibco), N2 supplement (Gibco), 4 μg/mL heparin (Sigma), 20 ng/mL human EGF (PeproTech), 20 ng/mL human b-FGF (PeproTech), 20 ng/mL human PDGF AA and PDGF BB (Shenandoah). Cells cultured as tumor neurospheres were passaged every 1–2 weeks when appropriate. In preparation for inoculation, cells were collected and resuspended in Matrigel (Corning Incorporated, Corning, NY) at 1x10^5^ cells/μL.

### Embryo preparation and tumor cell inoculation

Fertilized Leghorn chicken eggs were purchased from Hoover’s Hatchery (Ames, IA) and the embryo was transferred to a plastic weigh boat on day 4 of embryonic development (D4) and incubated at 37°C. On day 9 of embryonic development (D9), the CAM membrane was scored using a sterile Q-tip to remove the top layer of the CAM epithelium and access to the vasculature. A very small amount of bleeding (<5μL) is observed immediately after the injury. A total of 1x10^6^ cells mixed with Matrigel (1x10^5^ cells/μL; 10μL total) was added onto the scored CAM. One tumor was implanted per embryo. Chick embryos were gently placed back into the incubator and maintained 37°C over the course of the experiment.

### Drug treatment

Alisertib (MedChemExpress, Monmouth Junction, NJ), bortezomib (MedChemExpress), panobinostat (MedChemExpress), ponatinib (MedChemExpress), MSN1-Leu (synthesized in our lab, [20]) were sterilely prepared and dissolved in 0.5% DMSO and sterile PBS. Bevacizumab (SelleckChem) is water soluble, and was directly dissolved in sterile PBS. Drugs were administered by pipetting 15μL of drug solution directly on top of (but not contacting) the CAM tumor on embryonic development day 11, 13, 15. Each experiment contained 1–5 biological replicates per treatment group (at time of ultrasound) and there were at least three experimental replicates for each drug (n = 3–24 per drug treatment). Vehicle treatments (0.5% DMSO in sterile PBS) from all drug experimental replicates were combined to form one large vehicle group for further analysis (DIPGXIIIp* n = 38, DIPGIV n = 19).

### Radiation

CAM tumor irradiation was administered using the Precision X-RAD SmART irradiator platform capable of delivering image guided radiation therapy (Precision X-ray, North Branford, CT). 3D target localization was done using 3D cone beam computed topography (CBCT) and stage positioning was adjusted in 3 planes. Therapeutic radiation (2 grey) was delivered using a 10mm square collimator and 0.3 mm Cu filtration on embryonic development day 13.

### Ultrasound and tumor harvest

All tumors underwent 3-D ultrasound imaging on day 16 post-fertilization (D16) as described by Huang et al. [21]. Briefly, ultrasound imaging was performed with a Vevo 3100 high-frequency imaging system (FUJIFILM VisualSonics Inc., Toronto, Canada) and MX700 50MHz linear array transducer. Coupling gel (Aquasonic 100, Parker Laboratories Inc., Fairfield, NJ) was applied to the surface of the transducer transmitting in RF-power doppler mode. The transducer was centered above the tumor mass. The transducer incrementally stepped through the tumor volume in 0.25mm increments. Volume and vascularity analysis were done using VevoLab software (FUJIFILM VisualSonics Inc., Toronto, Canada). A region of interest (ROI) was manually drawn to quantify Doppler IQ signals within the tumor for each from. The vasculature index was defined as the area of the blood vessel within the ROI divided by the total area of the tumor ROI: VI = A_vessel_/A_tumor_ where A_vessel_ is the vessel area and A_tumor_ the area of the ROI. Vascularity index represents a measurement of the vessel density of the tumor. After ultrasound imaging, tumors were harvested from CAM and stored in 4% PFA at 20°C overnight before being blocked in paraffin for microtome sectioning.

### DIPGXIIIp* rodent xenograft

All animal experiments were conducted in accordance with the NIH guidelines for the use of animals in research and approved by the Mayo Clinic Institutional Animal Care and Use Committee (Protocol Number: A00002592-17). Rodent brain samples were acquired from nude rats following intracranial (pons) injection of 200,000 DIPGXIIIp* cells per 2μL under aseptic surgical conditions. Animals were anesthetized with 2.5% isoflurane. The skull was exposed and the animal was placed in a stereotactic frame (World Precision Instruments). A small burr hole was made at the stereotactic coordinates: 1mm inferior to the lamdoid suture and 1mm lateral to the mid-sagittal plane. DIPGXIIIp* were injected using a 26-gauge 10μL Hamilton syringe (Bonaduz, Switzerland) at a depth of 6mm below the skull and at a rate of 0.5μL/min (2μL, 4 minutes total). The needle was removed and the scalp closed with wound clips. Animal condition was monitored daily and at moribund, animals were euthanized and their brains fixed in 4% PFA before being blocked in paraffin for cryostat sectioning.

### Immunohistochemistry (IHC)

Following deparaffinization and rehydration, samples underwent antigen retrieval and blocking. They were incubated with H3K27M (1:500, AbCAM #190631), H3K27me3 (1:100, Cell Signaling #9733) for one hour at room temperature. Then we used goat anti-rabbit secondary antibody before visualization by DAB reagent (Cell Signaling, Danvers, MA). Samples were counterstained with haematoxylin and imaged using a Nikon SMZ18 (Melville, NY) microscope.

### Statistical analysis

GraphPad Prism Version 9.0 was used for statistical analysis. Data was expressed as mean values ± SD. Differences between groups were analyzed using one-way ANOVA followed by Dunnett’s multiple comparisons test. The level of significance was P<0.05.

## Results

### Patient-derived DIPG tumors graft well in the CAM model

**Fig 1:**
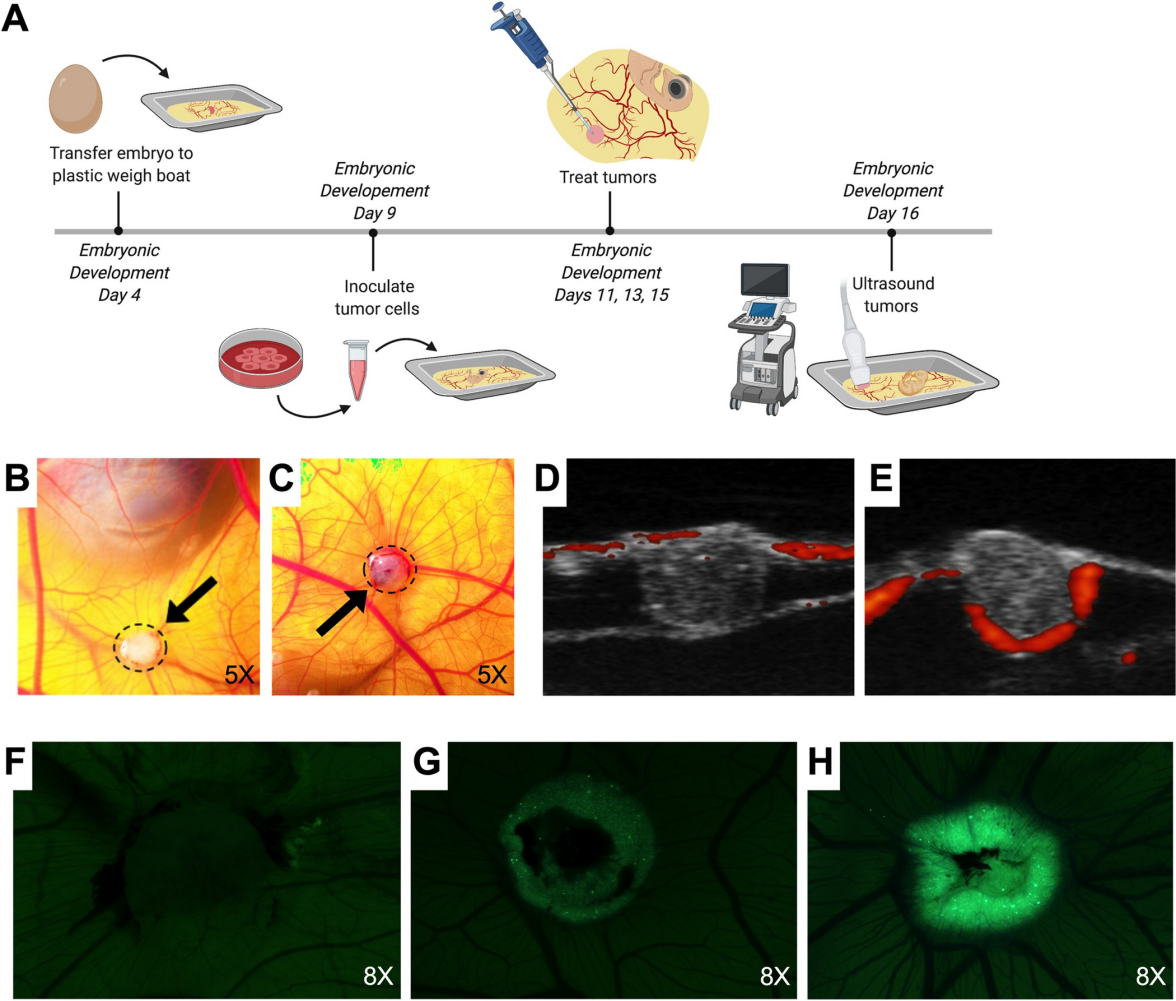
DIPG xenograft tumors are produced in the CAM model following inoculation with patient-derived cell lines. (A) Experimental procedures follow the corresponding timeline where 1x10^6^ DIPG cells are inoculated onto the CAM on embryonic development day 9. DIPGIV (B) and DIPGXIIIp* (C) tumors (dashed perimeter and black arrow) are visible within 48 hours of inoculation. Ultrasound visualization using RF Power Doppler Mode of DIPGIV (D) and DIPGXIIIp* (E) tumors reveals that the tumors grow below the CAM surface with blood flow visible around the edges of tumor and within CAM (red fluorescence: active blood flow). DIPGIV tumor growth can be appreciated through visualization of increased GFP signal of tagged tumor cells from day 11 (F) to day 13 (G) to day 15 (H).

The CAM model is dependent on the viability of the chicken embryos. While transfer of the embryo from the egg to the plastic weigh boat on embryonic development day 4 (Fig 1A) did not significantly impact embryo survival, general viability and fertilization rate was variable, ranging from 20% - 90%. Surviving embryos were with tumors inoculated on day 9 (Fig 1A) with an engraftment rate of 95%. On day 11, 48 hours after DIPGXIIIp* (H3.3K27M) and DIPGIV (H3.1K27M) tumor cell inoculation, visible spheroidal tumors were observed with DIPGXIIIp* DIPGIV (Fig 1B) and DIPGIV (Fig 1C). Survival of the embryos throughout the experimental timeline (D9 through D16) varied, but generally between 40–60% of tumors survived until day 16. Tumor development can be observed through ultrasound transmitting in RF Doppler mode. Imaging on day 16 showed that the majority of the tumor grows below the external surface in both DIPGXIIIp* (Fig 1D) and DIPGIV (Fig 1E). Also appreciable on ultrasound is tumor invasion into the CAM as well as blood flow (red fluorescence). As a secondary measure to observe tumor engraftment and growth, GFP expression was monitored in DIPGIV CAM tumors. Notable increases in expression can be appreciated from day 11 (Fig 1F) to day 13 (Fig 1G) and to day 15 (Fig 1H).

### DIPG CAM tumors maintain genetic and epigenetic phenotype of native DIPG tumor

**Fig 2:**
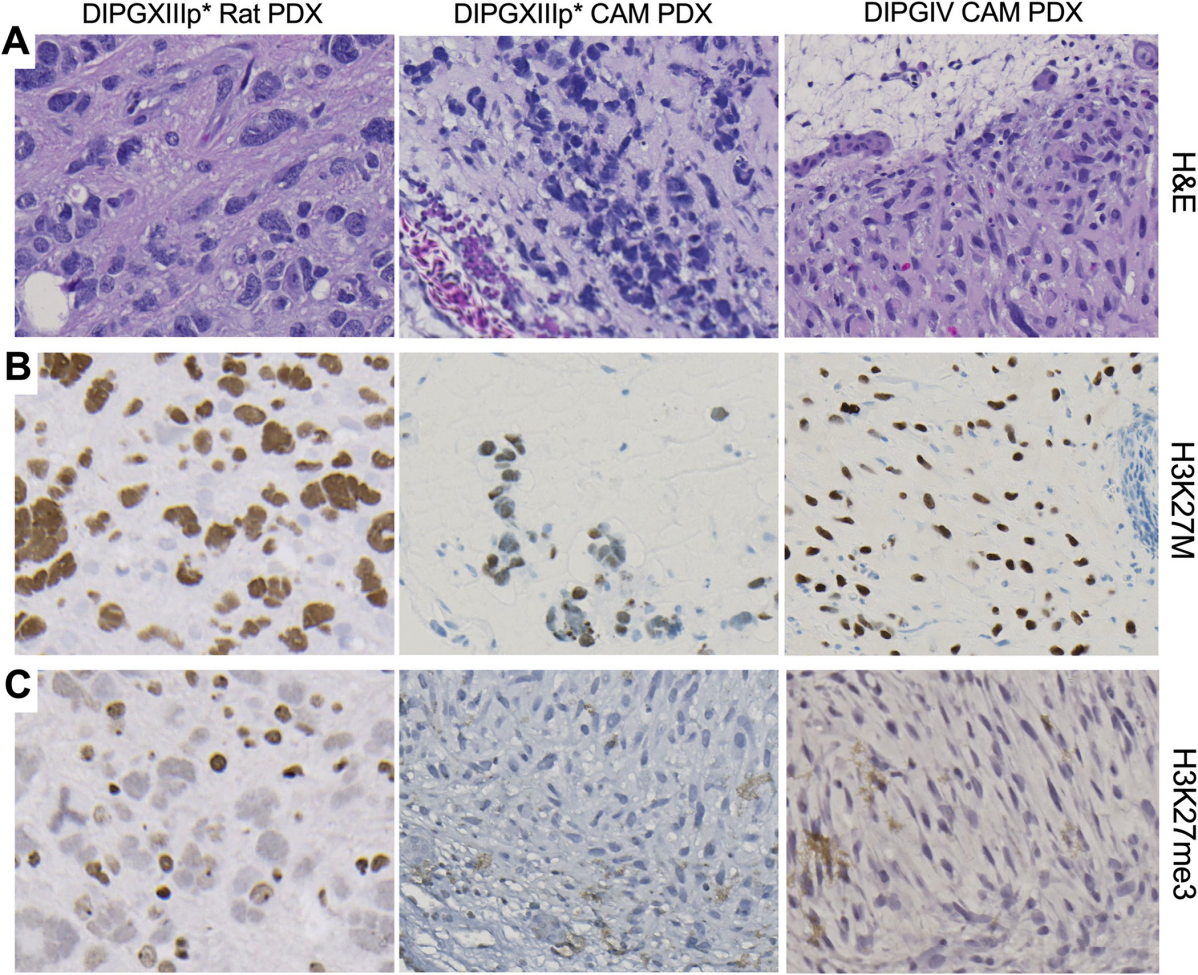
DIPG CAM tumors maintain molecular features of native tumor and rodent xenografts. (A) Hemoxylin and Eosin (H&E) staining of DIPGXIIIp* rodent, DIPGXIIIp* CAM and DIPGIV CAM tumors show tumor cell growth and invasion. Immunohistochemistry (IHC) staining shows positive H3K27M mutation status (B) and reduction of H3K27 trimethylation (C) for all tumors. All images were taken at 20X magnification.

To verify that patient-derived CAM tumors retain the phenotype of the native DIPG tumor, we used immunohistochemistry (IHC). First, analysis of Hemoxylin and Eosin (H&E) staining confirmed presence of tumor cells for both DIPGXIIIp* (Fig 2A, middle panel) and DIPGIV (Fig 2A, right panel). The infiltrative and diffuse invasion of CAM tumor cells is similar to what is observed in our rodent DIPGXIIIp* xenograft (Fig 2A, left panel). Additionally, characterization of DIPG relies on H3K27M mutation status and reduction in H3K27 trimethylation (me3), per the literature [12, 22, 23] and the World Health Organization [1]. The H3K27M mutation as well as reduction in H3K27me3 was shown in the original tissue samples from which the cell lines are derived from as well as in the derived cell lines [19]. Correspondingly, we show that the DIPGXIIIp* and DIPGIV CAM tumors maintain their positive H3K27M mutation status similar to the established DIPGXIIIp* rodent xenograft (Fig 2B). Similarly, the hallmark reduction in H3K27me3 is observed in the CAM, akin to rodent xenograft tumors (Fig 2C).

### CAM model as a potential xenograft platform for DIPG drug testing

**Fig 3:**
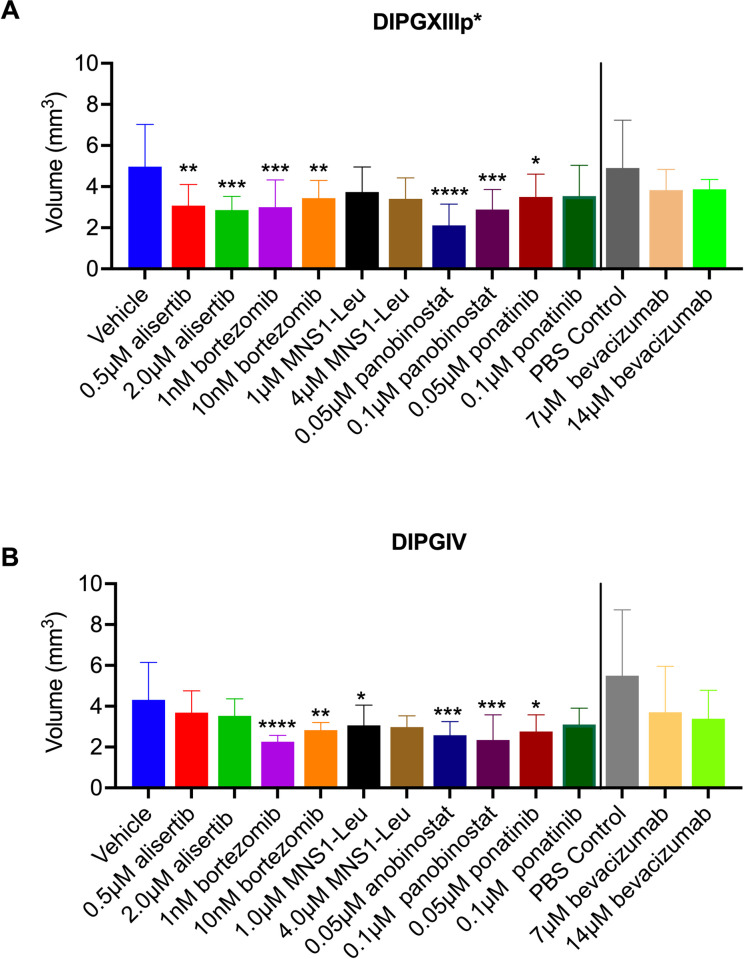
Volumetric analysis of DIPGXIIIp* and DIPGIV CAM tumors following drug treatment. DIPGXIIIp* (A) and DIPGIV (B) CAM tumors were treated with alisertib, bortezomib, MSN1-Leu, panobinostat, ponatinib and bevacizumab on embryonic development days 11, 13 and 15. Tumor volume was determined following 3-D ultrasound on embryonic development day 16. Vehicle is 0.5% DMSO in sterile PBS. PBS control is 100% sterile PBS. Differences were determined with one-way ANOVA and Dunnett’s test for multiple comparisons where P<0.05 is considered significant. P<0.05 = *, P<0.01 = **, P<0.001 = ***, P<0.0001 = ****.

To test the feasibility for drug screening in DIPG CAM tumors, we assessed tumor volume with 3-D ultrasound following topical treatment with different drugs previously identified in our laboratory and by others [24] by *in vitro* drug screens. In the H3.3K27M tumor model, DIPGXIIIp*, CAM tumors showed statistically significant decreases in tumor volume following treatment with 0.5μM and 2μM alisertib, 4μM MSN1-Leu, 0.5μM panobinostat, and 0.05μM and 0.1μM ponatinib (Fig 3A). DIPGXIIIp* CAM tumors did not show a statistically significant decrease in tumor volume following treatment with 1nM or 10nM bortezomib (p = 0.29, p = 0.91, respectively) or 1μM MSN1-Leu (p = 0.08) (Fig 3A). Since bevacizumab is water soluble, DIPGXIIIp* CAM tumors treated with 7μM or 14μM bevacizumab were compared to a PBS-treated control and the differences were not significant (p = 0.23, p = 0.39, respectively) (Fig 3A).

These compounds were assessed in a second DIPG cell line, DIPGIV, which contains a H3.1K27M mutation. DIPGIV CAM tumors showed statistically significant decreases in tumor volume following treatment with 1nM and 10nM bortezomib and 0.05μM and 0.1μM panobinostat (Fig 3B). There was no statistically significant decreases in tumor volume following treatment with 0.5μM or 2μM alisertib (p = 0.56, p = 0.41, respectively), 7μM or 14μM bevacizumab compared to PBS-treated control (p = 0.49, p = 0.46, respectively), 1μM or 4μM MSN1-Leu (p = 0.07, p = 0.17, respectively), or treatment with 0.05μM or 0.1μM ponatinib (p = 0.09, p = 0.22, respectively) (Fig 3B).

### CAM as a model to study anti-angiogenic effects in DIPG tumors

**Fig 4:**
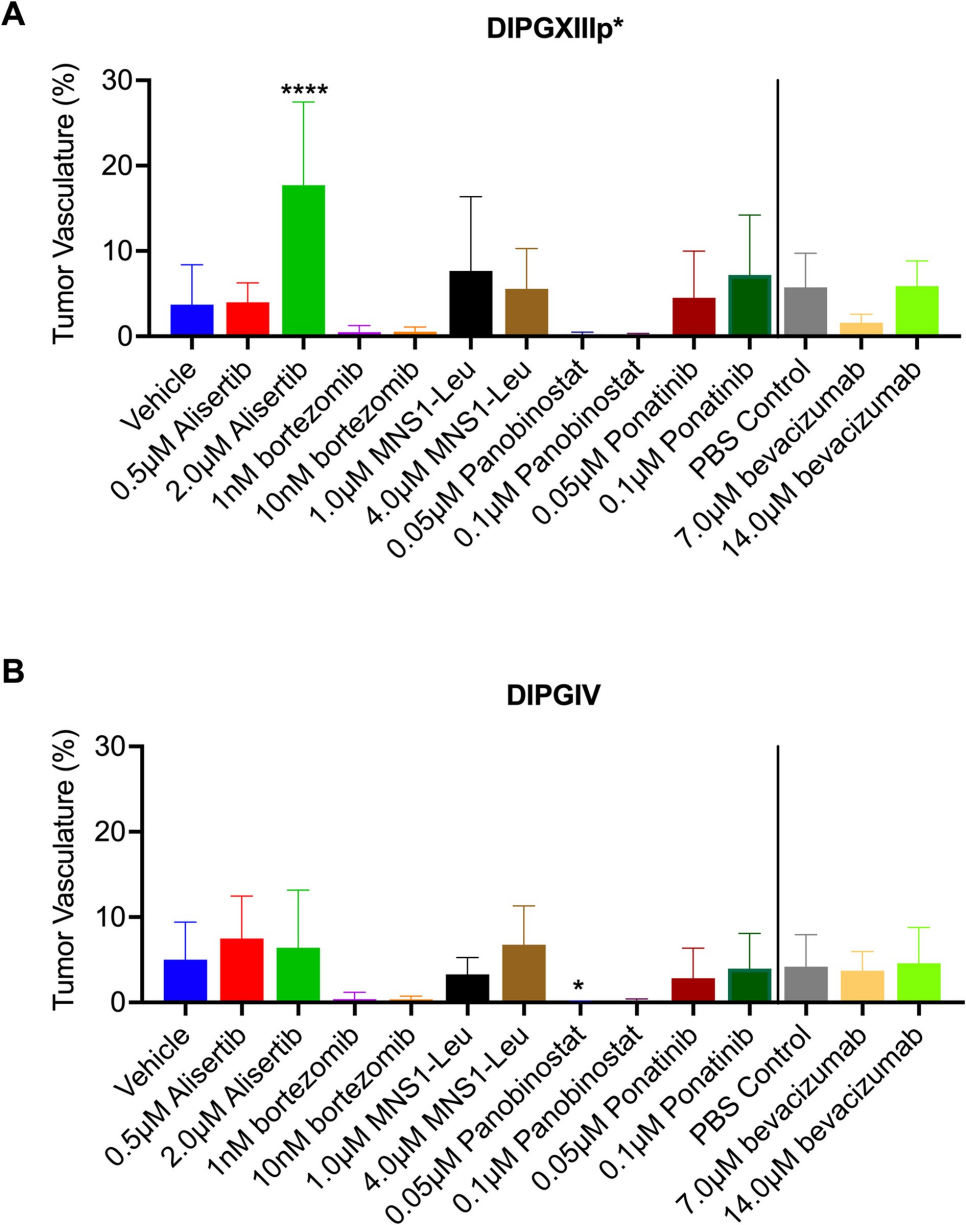
Analysis of tumor vascularity of DIPGXIIIp* and DIPGIV CAM tumors following drug treatment. DIPGXIIIp* (A) and DIPGIV (B) CAM tumors were treated with alisertib, bortezomib, MSN1-Leu, panobinostat, ponatinib and bevacizumab on embryonic development days 11, 13 and 15. Tumor vascularity was assessed following 3-D ultrasound on embryonic development day 16. Vehicle is 0.5% DMSO in sterile PBS. PBS control is 100% sterile PBS. Differences were determined with one-way ANOVA and Dunnett’s test for multiple comparisons where P<0.05 is considered significant. P<0.05 = *, P<0.01 = **, P<0.001 = ***, P<0.0001 = ****.

We sought to determine the feasibility of the CAM model to study DIPG angiogenesis. Tumor vascularity was assessed with 3-D ultrasound power doppler technology following drug treatments. A statistically significant increase in tumor vascularity was observed in DIPGXIIIp* CAM tumors following treatment with 2μM alisertib (Fig 4A). A statistically significant decrease in DIPG IV CAM tumors was observed following treatment with 0.05μM and 0.1μM panobinostat (Fig 4B). No other significant changes in vasculature were found in DIPGXIII CAM tumors treated with 0.5μM alisertib (p = 0.7149), 7μM or 14μM bevacizumab (p = 0.08, p = 0.99, respectively), 1nM or 10nM bortezomib (p = .43, p = 0.29, respectively), 1μM or 4μM MSN1-Leu (p = 0.75, p = 0.99, respectively), 0.05μM or 0.1μM panobinostat (p = 0.87, p = 0.71, respectively) and 0.05μM or 0.1μM ponatinib (p = 0.80, p = 0.77, respectively) (Fig 4A). No significant changes in tumor vascularity were found in DIPGIV CAM tumors following treatment with 0.5μM and 2μM alisertib (p = 0.41, p = 0.76, respectively), 7μM and 14μM bevacizumab (p = 0.97, p = 0.98, respectively), 1nM and 10nM bortezomib (p = 0.74, p = 0.68, respectively), 1μM and 4μM MSN1-Leu (p = 0.52, p = 0.72, respectively) or 0.05μM and 0.1μM ponatinib (p = 0.44, p = 0.82, respectively) (Fig 4B).

### CAM model as a xenograft model for DIPG radiation therapy

**Fig 5:**
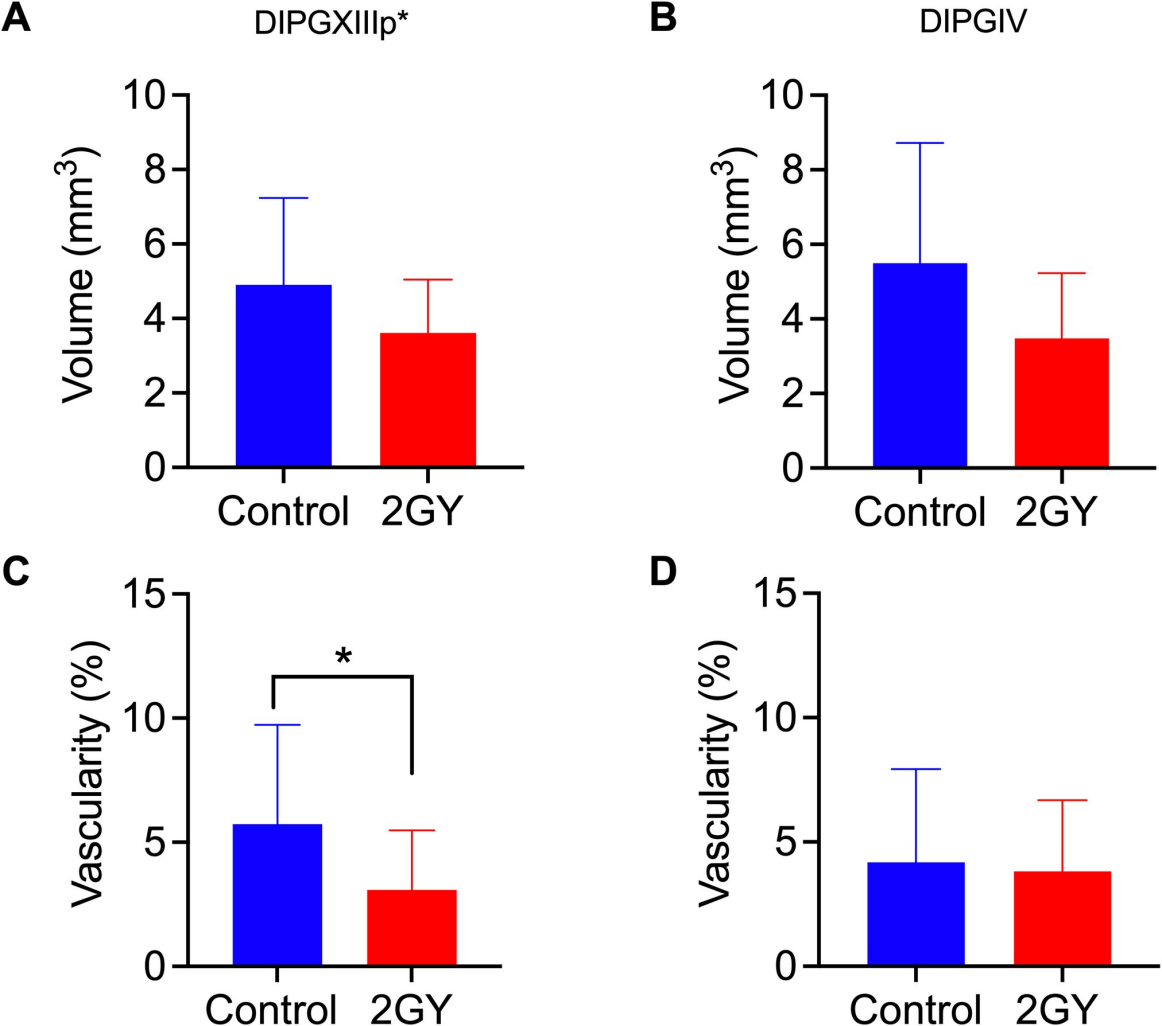
Volumetric and vasculature analysis of DIPG CAM tumors. DIPGXIIIp* and DIPGIV CAM tumors underwent a single dose of 2Grey radiation treatment on embryonic development day 13 and were analyzed by 3-D ultrasound on embryonic development 16. DIPGXIIIp* and DIPGIV CAM tumors were analyzed for differences in volume (A, B, respectively) and vascularity (C, D, respectively). Differences were analyzed by Student’s T-Test where P< 0.05. * = P<0.05, ** = P<0.001, *** = P<0.0001.

The standard of care for DIPG is radiation therapy (RT), although this is mostly palliative [2]. To confirm the CAM model could be used to evaluate response to RT we treated DIPXIIIp* and DIPGIV CAM tumors with a single dose of 2 Grey targeted radiation therapy and then assessed tumor volume and vascularity by 3-D ultrasound 72 hours later. There was no statistically significant decrease in tumor volume in DIPGXIIIp* tumors (p = 0.07) (Fig 5A) or DIPGIV tumors (p = 0.10) (Fig 5B). There was a significant decrease in tumor vascularity following RT in DIPGXIIIp* tumors (Fig 5C), but not in DIPGIV tumors (p = 0.82) (Fig 5D).

## Discussion

DIPG is an aggressive and lethal pediatric brain tumor for which no effective therapy has been elucidated. While recent breakthroughs in the molecular understanding of DIPG tumors have led to potential novel therapeutic targets, *in vivo* testing requires significant resources and time. The chicken embryo CAM model is a timely and low-cost intermediate xenograft model that could be utilized to bridge the *in vitro-in vivo* gap more efficiently [18]. Here, we established that patient-derived DIPG tumor cells can be inoculated on to the CAM with tumor formation within 48 hours. These tumors recapitulate native DIPG growth and histology and can be used evaluate responses to treatment.

The CAM model is well established as an experimental platform for studying other cancers including pancreatic [25], renal cell carcinoma [21] and urothelial carcinoma [17]. It has several key advantages, including a naturally immunodeficient state which makes engraftment of patient-derived tumors possible [18]. Furthermore, the CAM model is low-cost and extremely time-efficient. We found tumors are detectable with two days of DIPG cell inoculation and treatment assessment takes a maximum of eight days post inoculation, while murine models can take months to simply grow a tumor. The CAM model also comes with less ethical concerns and are not subject to IACUC regulation since the NIH does not consider avian embryos to be live vertebrates.

Previous studies have assessed the CAM model as a platform for drug testing in a variety of cancers including those mentioned above. To show that pharmacotherapy can be assessed in our DIPG CAM model, we selected drugs from previously published drug screens [26] and drugs screens done in our lab (unpublished) that were previously shown to be cytotoxic *in vitro*. Our study showed that DIPG CAM tumor response to pharmacotherapy can be monitored and assessed by 3-D ultrasound following topical drug application. This suggests that the CAM model provides a reliable and feasible model for assessing novel pharmacotherapies for DIPG, but is not without limitations. First, effectiveness of the topical application of drug solutions is dependent on drug penetration of the CAM into the tumor cells and the associated pharmacokinetics. Also, drug testing in the CAM model, particularly by topical administration, does not allow for evaluation of blood-brain-barrier (BBB) penetration, which is critical in developing effective therapy for this disease as DIPG is known to have an intact BBB. However, with a number of direct delivery methods, such as convection-enhanced delivery, topical administration may be beneficial as it avoids first pass metabolism and the need for bypassing the BBB which could be useful in selecting compounds for direct delivery testing in murine models. Second, our study utilized two patient-derived DIPG cell lines, DIPGXIIIp*, a H3.3K27M (*H3F3A)* mutated tumor and DIPGIV, a H3.1K27M (*HIST1H3B/C)* mutated tumor. Prior studies have established clinical and prognostic differences based on mutation origin and it might be possible that differences in responses to therapy observed could be attributed to the different mutations, but further studies in additional cell lines would be needed to confirm this [5, 27].

Prior studies have also assessed tumor angiogenesis via the CAM model [21, 28]. However, DIPG is generally considered a non-angiogenic tumor due to its general lack of contrast enhancement on MRI imaging at presentation [29]. Our data supports this claim as tumor vasculature was rarely above 10%. In comparison, renal cell carcinoma, a highly vascular tumor, has a tumor vascular index around 42% [21]. The low rate of neovascularization may explain the lack of a significant difference in tumor volume and tumor vasculature that we observed following treatment with bevacizumab, an established VEGF monoclonal antibody. Extremely low rates of tumor vascularization were observed following with panobinostat and bortezomib, although this was only significant following treatment of 0.05μM of panobinostat in DIPGIV CAM tumors (Fig 4B). The mechanism of action for these drugs does not normally contribute to significantly lower rates of tumor vascularization that we observed. It is possible that these drugs had some off-target effects that may have effected neovascularization within the engrafted CAM tumor and further studies in the future could help elucidate this observation further. However, a general absence of prominent angiogenesis in all DIPG CAM tumors did not lead to poor rates of implantation and tumor growth as seen by Capdevielle et al. [30] and therefore did not require overexpression of VEGF for implantation, maintaining the natural biology of DIPG tumors.

A majority of DIPG patients receive radiation therapy [2, 31] and is an important consideration in the development of novel treatment strategies in DIPG. To the best of our knowledge, no study to date has attempted RT on CAM tumors. We were able to treat the DIPG CAM tumors with targeted radiation and saw decreases in tumor volume in both cell lines compared to no radiation controls, but the differences were not statistically significant. The ability to assess response to RT in the CAM model may lend well to future studies to assess radiosensitivity differences in DIPG tumors or combination therapies with various radiosensitizers. However, it is difficult to assess the long-term effects of radiation treatment due to the short-term nature the CAM assay.

The CAM model itself has several key limitations. The use of chicken embryos as a model organism can be challenging because embryo viability is largely variable (20–90%) especially depending on sudden meteorological changes, like extreme temperatures swings, which leads to lower rates of fertilization as well as lower survival of remaining embryos. The study is limited by its strict timeframe as the eggs are only viable until day 17 of embryonic development which can be potentially problematic when trying to observe response to treatment depending on the mechanism and also makes survival studies impossible. Finally, we used 3-D ultrasound to assess tumor volume and vasculature, however, this technique is limited because the chicken embryos can only survive a single ultrasound. Thus, multiple ultrasounds for monitoring treatment responses are not possible. Despite these limitations, the CAM model is neither technically challenging nor time consuming or expensive. Therefore, it can be readily used as a model for bridging the *in vivo–in vitro* gap. One example of for using the DIPG CAM model could be refining large scale *in vitro* drug screens may result in a large number of drug hits. Conducting *in vivo* murine studies with every drug hit would be impossible and often, in the case of DIPG, cell culture results do not always readily translate to the murine models. Although this is beyond the scope of this study, the CAM model could be used in helping select agents for consideration for longer and more expensive in vivo murine testing.

In conclusion, we established the CAM model as a potentially convenient, low-cost xenograft model to study treatment approaches for DIPG. We showed that DIPG tumors grown on the CAM membrane mimic the native characteristics of this disease and evaluated the propensity for a variety of treatment modalities including pharmacotherapy and radiation in this study. Further larger scale studies will be necessary to assess the practicality of this DIPG model in the research pipeline.

## Supporting information

S1 Data (XLSX)

## Abbreviations

CAM: chorioallantoic membrane
DIPG: diffuse intrinsic pontine glioma
RT: radiation therapy
